# Two-Week Wait Gastrointestinal (GI) Cancer Pathway: A Single Tertiary Centre Experience During the COVID-19 Pandemic

**DOI:** 10.7759/cureus.36857

**Published:** 2023-03-29

**Authors:** Tareq Al Saoudi, Suchita Bahri, Farah Khasawneh, Neil Bhardwaj, Giuseppe Garcea

**Affiliations:** 1 Department of Hepato-Pancreato-Biliary (HPB) Surgery, University Hospitals of Leicester National Health Service (NHS) Trust, Leicester, GBR

**Keywords:** gi cancer, two week wait pathway, general practice, surgical oncology, covid-19

## Abstract

Background: This article investigated the impact of COVID-19 on the two-week wait referral pathway at the University Hospitals of Leicester NHS Trust. The conversion rate of these referrals was also explored as an indicator of the appropriateness of referrals from primary care.

Methods: Two-week wait referrals to the Cancer Centre of the University Hospitals of Leicester NHS Trust from 2018 to 2020 were collected for upper gastrointestinal (UGI), lower gastrointestinal (LGI), and hepato-pancreato-biliary (HPB) surgery. The confirmed cancer cases out of these referrals were also recorded. Additionally, the outcomes of the multidisciplinary team (MDT) meetings for all patients discussed in June 2018, 2019, and 2020 were collected, and their staging and treatment data were examined.

Results: The number of two-week referrals decreased in 2020 compared to the previous two years across the three specialities. This was more pronounced in April, with a reduction of over 50%. The conversion rate of these referrals increased in 2020 compared to 2018 and 2019 among all three specialities. The increase in conversion rate was statistically significant for LGI referrals (2018 vs 2020 p = 0.0056; 2019 vs 2020 p = 0.0005). There was no significant difference in the MDT outcome across the three specialities.

Conclusion: Two-week wait remains a cornerstone pathway in the management of patients with suspected cancer in the National Health Service. The COVID-19 pandemic appeared to have reduced inappropriate referrals, as evidenced by the increased conversion rate. This did not appear to negatively impact tumour staging and outcomes for those patients who were referred on the pathway.

## Introduction

The National Health Service (NHS) in the United Kingdom (UK) underwent an unprecedented shift in service delivery during 2020 to focus its resources on combatting the COVID-19 pandemic. An overburdened healthcare system, along with the impact of national lockdown and public apprehension about accessing healthcare facilities, resulted in a delay in the recognition, referral, diagnosis, and treatment of non-COVID-19-related diseases such as cancer pathways [1].

It is widely acknowledged that diagnostic delays can lead to delays in cancer detection, with a resultant worse prognosis and survival rate due to “stage migration” [2,3]. A special NHS pathway called “Two-Week Wait” referrals was created to track and help prevent delays in diagnosis and treatment. This pathway requires all suspected cancer cases to be evaluated by a specialist within two weeks of presentation.

Multiple reports have described the profound impact of the COVID-19 pandemic on the management of cancer patients, including delayed diagnosis due to prolonged waiting times for investigations (including two-week wait referrals) and delays in the commencement of treatment and follow-up [4]. A report from Macmillan Cancer Support recently found that there are over 50,000 cases of missed cancer diagnoses within the UK. This has raised concerns that cancer patients are at risk of becoming the “forgotten C” of the COVID-19 crisis [5]. The massive drop in the two-week wait referrals, which was reported to be more than 70% during April-May 2020, has been identified as a major contributing reason for the abovementioned figure.

The present study investigated how the COVID-19 pandemic affected the two-week wait referral pathway at the University Hospitals of Leicester NHS Trust, focusing specifically on gastrointestinal tract cancers. Additionally, to address the issue of excess referrals by primary healthcare providers, the study examined confirmed cancer cases that resulted from these referrals. Confirmed cancer conversion rates were used as a marker.

## Materials and methods

A retrospective analysis of data gathered from the Cancer Centre of the University Hospitals of Leicester NHS Trust was conducted for the present study. This included all two-week wait referrals from 2018 to 2020 within the Trust for the specialities of upper gastrointestinal (UGI), lower gastrointestinal (LGI), and hepato-pancreato-biliary (HPB) surgery. Additionally, the confirmed cancer cases from these referrals were also noted. This allowed for a conversion rate to be calculated.

The outcomes of multidisciplinary team (MDT) meetings for all patients who were discussed in June 2018, 2019, and 2020 were collected. Further data regarding their staging and treatment were obtained from the electronic records of the University Hospitals of Leicester NHS Trust.

Statistical analysis

All data were entered into an Excel database (Microsoft, Redmond, Washington, USA), and analysis was performed using GraphPad Prism for Windows, version 8.1.2 (GraphPad Software, Inc., California, USA). Comparisons amongst groups and over time were performed using the analysis of variance (ANOVA) test and non-parametric t-test. P values <0.05 were considered statistically significant.

## Results

**Figure 1:**
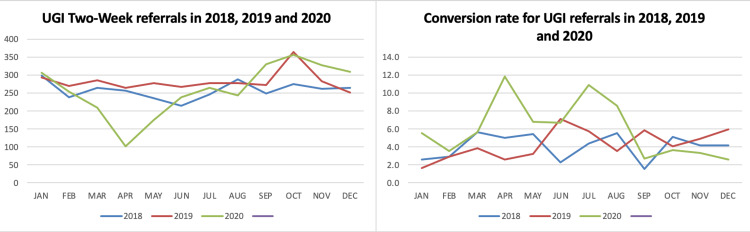
Two-week referrals and conversion rate for UGI referrals in 2018, 2019, and 2020 UGI: upper gastrointestinal

**Figure 2:**
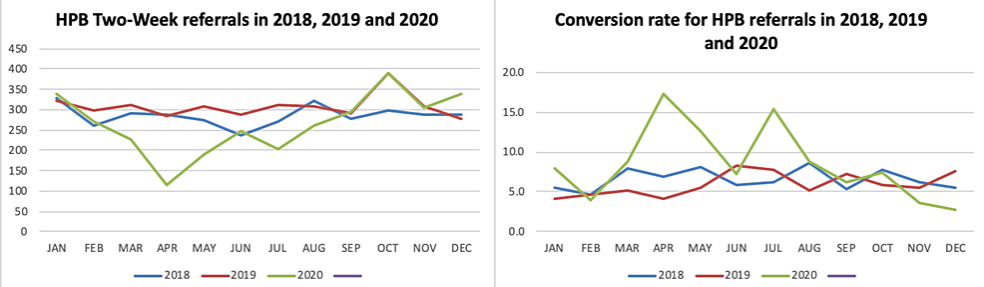
Two-week referrals and conversion rate for HPB referrals in 2018, 2019, and 2020 HPB: hepato-pancreato-biliary

**Figure 3:**
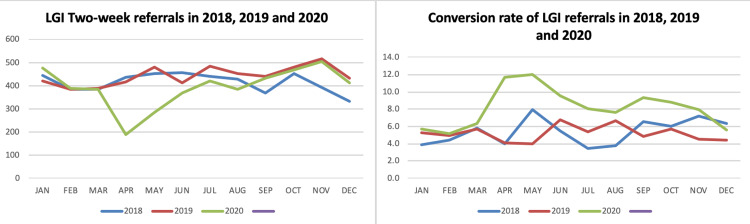
Two-week referrals and conversion rate for LGI referrals in 2018, 2019, and 2020 LGI: lower gastrointestinal

The number of two-week wait referrals in all three surgical specialities decreased in 2020. This was more obvious in April 2020, when the drop was more than 50% (Figures 1-3). Interestingly, compared to the previous two years, the number of confirmed cancer cases from these referrals increased in 2020 (Tables 1-3). UGI cancer cases were detected at a rate of 5.4% in 2020, compared to 4.3% and 4.1% in 2019 and 2018, respectively (Table 1). In HPB cancer cases, the conversion rate increased from 6.6% in 2018 and 5.9% in 2019 to 7.6% in 2020 (Table 2). The same pattern was observed in LGI cancer as well, with the rate of confirmed cases increasing from 5.3% in 2018 and 5.1% in 2019 to 7.9% in 2020 (Table 3).

**Table 1:** UGI two-week referrals and confirmed cancer cases in 2018, 2019, and 2020 UGI: upper gastrointestinal

	2018		2019		2020	
	Referrals	Confirmed	Referrals	Confirmed	Referrals	Confirmed
JAN	300	8	293	5	308	17
FEB	238	7	271	8	254	9
MAR	266	15	285	11	210	12
APR	258	13	265	7	101	12
MAY	237	13	278	9	176	12
JUN	214	5	267	19	239	16
JUL	247	11	278	16	266	29
AUG	289	16	277	10	243	21
SEP	250	4	273	16	332	9
OCT	275	14	365	15	356	13
NOV	263	11	284	14	329	11
DEC	264	11	251	15	310	8
SUM	3101	128	3387	145	3124	169
Conversion rate	4.13%		4.28%		5.41%	

**Table 2:** HPB two-week referrals and confirmed cancer cases in 2018, 2019, and 2020 HPB: hepato-pancreato-biliary

	2018		2019		2020	
	Referrals	Confirmed	Referrals	Confirmed	Referrals	Confirmed
JAN	330	18	320	13	338	27
FEB	262	12	299	14	272	11
MAR	291	23	313	16	228	20
APR	287	20	286	12	116	20
MAY	274	22	309	17	191	24
JUN	236	14	289	24	246	18
JUL	272	17	311	24	202	31
AUG	323	28	307	16	259	23
SEP	277	15	292	21	294	18
OCT	298	23	390	23	388	29
NOV	288	18	307	17	303	11
DEC	289	16	277	21	337	9
SUM	3477	226	3700	218	3174	241
Conversion rate	6.59		5.89		7.59	

**Table 3:** LGI two-week referrals and confirmed cancer cases in 2018, 2019, and 2020 LGI: lower gastrointestinal

	2018		2019		2020	
	Referrals	Confirmed	Referrals	Referrals	Referrals	Confirmed
JAN	445	17	419	22	477	27
FEB	383	17	383	19	388	20
MAR	382	22	386	22	382	24
APR	434	17	416	17	189	22
MAY	452	36	481	19	284	34
JUN	455	25	413	28	368	35
JUL	440	15	485	26	421	34
AUG	429	16	450	30	383	29
SEP	368	24	440	21	431	40
OCT	450	27	478	27	467	41
NOV	391	28	514	23	503	40
DEC	332	21	430	19	410	23
SUM	4961	265	5295	273	4703	369
Conversion rate	5.34%		5.16%		7.85%	

In 2020, the conversion rate increased in a non-uniform manner. The highest referral rates for HPB and UGI cancers were observed in April 2020 and July 2020, with the rates for the rest of the months being equivalent to those of 2018 and 2019 (Tables 1-2 and Figures 1-2). In April and May 2020, the rate of detection for LGI referrals grew dramatically. The detection rate gradually fell for the rest of the year, but it remained more significant than those of the previous two years (2018 vs 2020 p = 0.0056; 2019 vs 2020 p = 0.0005) (Table 3 and Figure 3).

Although the number of patients discussed in each MDT was less in 2020 compared to 2019 and 2018, there was no significant difference in the MDT outcome and staging across the three specialities (Tables 4-6).

**Table 4:** UGI MDT outcome for June 2018, 2019, and 2020 UGI: upper gastrointestinal; MDT: multidisciplinary team

	2018	2019	2020
Neoadjuvant	1	7	3
Surgery	3	4	1
Adjuvant	2	1	0
Palliative	2	7	3
Stage T1	0	0	1
Stage T2	2	1	0
Stage T3	1	8	3
Stage T4	0	3	2
Stage N0	2	0	0
Stage N1	1	7	2
Stage N2	0	3	2
Stage N3	0	2	2
Total	9	14	6

**Table 5:** HPB MDT outcome for June 2018, 2019, and 2020 HPB: hepato-pancreato-biliary; MDT: multidisciplinary team

	2018	2019	2020
Neoadjuvant	0	4	3
Surgery	5	2	3
Adjuvant	2	3	1
Palliative	5	5	4
Total	11	14	11

**Table 6:** LGI MDT outcome for June 2018, 2019, and 2020 LGI: lower gastrointestinal; MDT: multidisciplinary team

	2018	2019	2020
Stage T1	2	2	1
Stage T2	4	4	2
Stage T3	12	11	10
Stage T4	4	5	6
Stage N0	12	5	6
Stage N1	7	10	6
Stage N2	5	6	5
Stage N3	5	6	4
M1	3	5	3
Total	23	22	19

## Discussion

It is assumed that a healthcare system with good primary care, such as the NHS, has a longer referral time to secondary healthcare providers, resulting in patients with cancer presenting at an advanced stage, with a poor prognosis [6]. This delay was thought to be one of the reasons for the UK’s lower cancer survival rate when compared to the rest of Europe [7,8]. To counter this, the NHS implemented the urgent suspected cancer pathway or the two-week wait referral. This pathway requires all referrals to be examined within 14 days by a specialist and the first step in management to be started within 62 days if cancer is detected [9].

Although this pathway provided patients with immediate access to healthcare, it placed a significant burden on secondary and tertiary healthcare facilities to review and investigate these referrals within the two-week time frame, especially given the rapid increase in the number of these referrals over the last decade. The frequency of urgent referrals climbed by 145% between 2009 and 2019 according to a report published by the National Cancer Intelligence Network. Although the number of verified cancer cases increased by 61% over the same time period, it did not keep pace with the increase in referrals. As a result, the conversion rate of these two-week recommendations decreased from 10.8% in 2009 to 7.1% in 2019 (Table 7) [10].

**Table 7:** Urgent referrals, confirmed cancer cases, and conversion rate in England 2009-2019

Year	No. of Referrals	Confirmed Cancer Cases	Conversion Rate
2018/19	2,219,821	157,987	7.12
2017/18	1,943,418	146,611	7.54
2016/17	1,862,929	141,785	7.61
2015/16	1,722,812	133,946	7.77
2014/15	1,545,614	126,630	8.19
2013/14	1,353,560	122,226	9.03
2012/13	1,215,758	114,943	9.45
2011/12	1,101,780	110,396	10.02
2010/11	999,635	103,019	10.31
2009/10	902,943	97,755	10.83

Multiple reports have raised concerns about the quality of referrals and the unnecessary use of this pathway because of the increase in the number of referrals and the corresponding decrease in the conversion rate [11-14]. The data from the University Hospitals of Leicester during the pandemic supports these worries. Despite a drop in referrals in 2020, the number of verified cancer cases was found to have risen, with the conversion rate of urgent referrals having improved significantly among the three specialities.

It is possible that the decrease in referrals corresponds to a reduction in “unnecessary” referrals on the two-week wait pathway, owing to the pandemic. This is supported to some extent by the observation that the number of cancer cases diagnosed did not decrease compared to 2019. However, it is important to note that the pandemic has potentially created a blanket time shift from the point of detection [15]. Though the conversion rate reflects a possible improvement, it does not, however, establish the stage and therefore the severity of the disease being diagnosed. This is a key point to consider whilst evaluating the impact of the pandemic on the disease population and their outcomes. As illustrated in Tables 4-6, all three MDTs saw fewer patients in June 2020, but the incidence of patients at later stages of the disease was comparable to the previous years.

There may still be more patients who are as yet undiagnosed, and their incorporation into the statistics could adversely influence these findings. Furthermore, this study dealt specifically with patients who presented to their GP and were placed on a two-week wait pathway; there are undoubtedly other patients who would have presented via other routes, such as emergency presentations (due to concerns regarding the pandemic or due to a reluctance to overburden the NHS). Finally, this study did not examine the impact of delays in treatment (e.g., surgery or chemotherapy) related to the reduction in capacity in 2020.

A detailed discussion of the factors driving the incremental step-up in referrals on the two-week wait pathway is beyond the scope of this study, although it can be ascribed to the following: First, the National Institute for Health and Care Excellence (NICE) modified the referral criteria in June 2015, lowering the referral threshold [16]. Second, primary care practices with a high referral rate have been linked to lower mortality; hence, GPs have recently been pushed to use this urgent approach [17]. Furthermore, the health secretary has advocated “naming and shaming” of GPs who fail to spot cancer, and rising reports of such instances in social media may have contributed to this increase [18].

There has been a steady increase in the number of two-week wait referrals, which has placed a strain on tertiary healthcare providers to meet the target. The pandemic has caused a massive backlog of non-urgent cases that must be dealt with. According to the president of the Royal College of Surgeons, there are more than five million patients waiting for procedures in the UK, the highest since modern records began [19]. With the current increase in referral rates and presumed “excessive referrals,” as well as the pandemic’s profound effect, the viability of the two-week pathway is debatable in terms of the increased number of clinics, endoscopy lists, and radiological investigations as well as the cost and workforce required to deal with these referrals [20].

## Conclusions

Whilst the two-week wait cancer referral pathway remains an important mechanism for providing quick access to specialist treatments to patients with probable cancer, it is now imperative to streamline this process. The findings of this study highlight the need for more robust and specific referral criteria for suspected surgical cancers. This can be achieved by working cohesively with primary care providers and assisting them with benchmarking, peer reviewing, and auditing their suspected cancer referrals in order to identify areas of improvement within the referral process. This could lead to increased diagnostic efficacy of the two-week wait referral pathway, potentially impacting the prognostic outcome of cancer patients in the UK.
